# *‘Incense is the one that keeps the air fresh’*: indoor air quality perceptions and attitudes towards health risk

**DOI:** 10.1186/s12889-024-20635-1

**Published:** 2024-11-14

**Authors:** Ashley Williams, Kayla Schulte, Diana Varaden

**Affiliations:** 1 School of Public Health, Imperial College London, London, UK; 2 MRC Centre for Environment and Health, Imperial College London, London, UK; 3 NIHR-HPRU Environmental Exposures and Health, School of Public Health, Imperial College London, London, UK

**Keywords:** Indoor air pollution, Indoor air quality, Household air pollution, Sensory perception, Air pollution health risk, Health disparities, Public health, Environmental health

## Abstract

**Background:**

Air pollution is of significant environmental and public health concern globally. While much research has historically focused on outdoor air pollution, indoor air pollution has been relatively under-explored despite its strong connection with health outcomes, particularly respiratory health. Studies on air pollution exposure mitigation consistently reveal a significant knowledge gap between the understanding of air pollution as a health risk among lay individuals and expert scientists. This study aimed to assess how people define and understand the concept of ‘clean air’ within their home setting.

**Methods:**

We adopted a mixed-methods approach which used a guided questionnaire designed to elicit both quantitative and qualitative data, collected as digital voice notes. The total sample (*n* = 40) comprised data from two socially different sites of science and non-science events. We compared whether the notion of clean air inside homes differs between these two different social contexts and how views and ‘sense’ of indoor air pollution are formed. The concept of ‘place’ facilitated fluidity in our explorative analysis. Insights allowed us to assess the extent to which context mediates individuals’ perceptions of indoor air pollution and attitudes towards health risk.

**Results:**

We found that individuals’ insights were embodied in repetitive day-to-day activities (e.g. cleaning and cooking). Three key themes emerged (1) *Stimulative Effects*, (2) *Contextual Conditions*, and (3) *Risk Attitudes*. Sensory perceptions such as sight, smell and temperature primarily motivated participants to assess air quality inside their homes. These perceptions were shaped by contextual conditions, influencing how individuals perceived their health risk and were subsequently motivated to spend personal time considering or seeking information about household air pollution, or improving their home air quality.

**Conclusions:**

Our insights revealed that social, geographical, and contextual factors play a crucial role in individuals’ understandings of indoor air pollution. These dimensions should be integrated into designs of effective public health risk communication strategies. Our findings highlight that common lay perceptions and practices intended to improve air quality may pose health risks. Therefore, risk communication about household air pollution must extend beyond objective information by considering contextual factors that shape how people interpret and respond to air quality issues.

**Clinical trial number:**

Not applicable.

**Supplementary Information:**

The online version contains supplementary material available at 10.1186/s12889-024-20635-1.

## Highlights

Limited research exists in the UK about public understanding of indoor air pollution within residential settings and its implication to public health.

The theoretical lens of ‘place’ identified sensory perception as a key motivator for participants in understanding indoor air quality within their homes.

Social, geographical and contextual dimensions are fundamental to population understanding of indoor air quality health risk within residential settings.

Current public health interventions may not be effective and meaningfully impactful because the messaging does not align with people’s sensory perceptions of indoor air quality.

Study findings can support the UK’s refinement of current practices surrounding indoor air pollution health risk mitigation.

**Supplementary Information:**

The online version contains supplementary material available at 10.1186/s12889-024-20635-1.

## Introduction

Air pollution (AP) occurs due to the accumulation of harmful substances in the atmosphere, leading to poor air quality that affects human health and the environment [1].

91% of the global population live in places exceeding the World Health Organization (WHO) guideline limits [2]. In the UK, air pollution is considered the largest environmental risk to public health, contributing to an actual equivalent of 36,000 premature deaths annually and health impact costs of £20 billion [3].

Tacit knowledge reinforces that indoor spaces, particularly our homes, offer safety and protection from air pollution. However, indoor air quality (IAQ), including inside homes, can be worse than outside due to the inherent heterogeneity in exposures linked to individual behaviours [4]. This is compounded by factors such as pollution infiltration, poor ventilation and combustion activities, leading to the accumulation of toxic pollutants indoors [5–7]. Common sources include repetitive household practices and activities such as cooking [8], cleaning using chemical and disinfectants products [9, 10], smoking tobacco, burning candles and incense [11, 12], occupying poorly ventilated buildings [13], as well as biological sources such as pet dander and mould [3]. A meta-analysis [14] on household air pollution (HAP) impacts found high positive association with health conditions including asthma in children and adults, lung cancer, chronic obstructive pulmonary disease, and cardiovascular mortality.

The 2022 report on air pollution by England’s Chief Medical Officer (CMO) [15] noted a general lack of indoor air pollution research and public understanding of health implication; adding, with improvement outdoors, the ‘problem’ is shifting indoors where very little is known about public understanding of air quality. This is underscored by modern-day living practices, particularly in global north industrialised regions, where evidence shows populations spend over 90% of their time indoors [16–18]. Relative to UK outdoors, acceptable concentration levels for indoor air pollution is less defined, and existing health-based advice issued by WHO and PHE lack statutory underpinnings [4]. A recent study [19] argues that existing standards that measure IAQ often lack clear metrics, including thresholds on people’s perceptions of IAQ. The study proposes a standard for how future studies can comparatively evaluate indoor smells and odours, for the purposes of moving beyond neutral odour as the ideal, but incorporating pleasant smells. Methodologically [20], a literature review of qualitative studies on air pollution risk perception found few exist, and even fewer using mixed methods. The study highlighted the importance of interdisciplinary tools in providing holistic understanding of environmental health patterns observed in quantitative research. Furthermore, the general tendency in existing air pollution literature and discussion is to overlap commercial and household pollution under the term ‘indoor’ air pollution [21].

Air Pollution studies on exposure mitigation [20, 22, 23, 24] consistently highlight significant differences in the understanding of air pollution as a health risk between lay individuals and expert scientists. For example, a study in the UK [24] examined demographic and psychosocial factors associated with adherence and non-adherence to health advice accompanying public air quality warning systems; they found frequent suboptimal adherence rates, and with demographic factors not consistently predicting adherence. The study also identified psychosocial adherence facilitators including knowledge of publicly accessible air quality information alerts and beliefs that one’s symptoms were due to air pollution. Barriers included perceived susceptibility, perceived lack of self-efficacy, and reliance on sensory cues. Another study [22] used health behaviour change theories to explore why policy measures designed to reduce the impact of air pollution exposure on the public’s health are often ineffective. They proposed a framework and argued for increasing both the personal representativeness of air quality data and increasing citizen involvement through public engagement as key simultaneous steps that can better support action for individual protections against air pollution exposure.

Our study went further in seeking to gain better understanding of how individuals represent their experiences of IAQ within their homes, where repetitive day-to-day practices can at any time generate air pollutants that may be harmful to health. Uncovering individuals’ perception of IAQ within their homes is an important part of effective and meaningful public health air pollution risk communication strategy. Therefore, by adopting a comparative approach to exploring how different groups from different social contexts habitually represent and make ‘sense’ of IAQ in contexts of ‘micro geographies’ within their homes, our mixed-methods study contributes to the limited knowledge in broader public understanding of household air pollution in the UK. Ultimately, public perceptions of household air pollution form the building blocks of attitudes towards health risk.

The notion of ‘clean air’ and concept of ‘place’ provides novel lens to explore public perception of HAP and attitudes towards health risk. IAQ within residential settings is complex [4]. Concentrations and pollution sources can vary [8, 25] substantially according to factors including individual habits [9, 11, 12, 26], socioeconomic deprivation [21, 27, 28], building structure and materials [13, 29] as well as outdoor-to-indoor air exchange [5].

This study aims to better understand how individuals living in West London, UK, perceive IAQ and health risk within their homes. We argue that reliance upon sensory cues to determine what constitutes clean air is invariably subjective and is characterised by distinctive contextual factors (‘place’) [20, 24, 30, 31]. The concept of ‘place’ [32] is leveraged to contextualise and situate individuals within their homes in order to uncover how understandings of ‘clean air’ materialise. This helps shift the focus onto the social and positional dimensions of HAP knowledge and health risk, which can be distilled and filtered out throughout the production of natural scientific information [33]. Ultimately, this approach sheds light on how HAP, as an environmental health risk, is not an objective reality; rather, HAP is understood socio-culturally, subsequently, embodied in perceptions and attitudes towards health risk.

## Conceptual framework ‘Place’: localising perceptions of HAP

‘Place’ [32] is key in understanding perceptions of HAP and attitudes towards health risk. This is demonstrated in non-representation theory which expanded the use of ‘place’ in socio-environmental research. Here, ‘place’ can be considered simultaneously as something that locates, as well as surrounds and contains. As such, ‘place’ can be fluid. Anderson et al. [32], point to others who characterise ‘place’ as a site’s ‘locale’, encapsulating the built and social context of community relations; which can also involve a distinctive worldview or way of life generally associated with that ‘place’. Therefore, while the word ‘place’ in principle is broadly recognised in the function of situating objects geographically, conceptually it can be a multi-pronged research tool with reanimating potential.

This study’s invocation of ‘place’ is in terms of both geographical and social context of the home. Doing so helps demonstrate the fluidity in individuals’ perspectives of what constitutes pollutants, mediated by lived experiences. As a conceptual framework ‘place’ is functional in situating and reproducing meaning through local discursive interactions. Bickerstaff and Walker (2003) previously made the argument for contextualising AP knowledge, suggesting that people’s encounters and ways of ‘knowing’ pollution, polluters, polluted places and polluted people is embodied and centrally embedded in ‘place’. This underscores the importance of localisation in interpreting perceptions by questioning the meaning of AP in social constructionism of AQ perceptions.

Our study extends the conventional conceptualisation of ‘place’ in AP public perceptions from the ‘place’ of scientific understandings outdoors (ambient air) [31, 34–36] and brings it into less studied social understandings of ‘place’ indoors (HAP), within distinctive contexts of individuals’ homes.

## Methods

### Study design

This study used mixed methods to explore perceptions of HAP and attitudes towards health risk from two contextually different sites in West London, UK, for comparative analysis. Site one (S1) was a science-technology context, while site two (S2) was a non-science-technology context. The guided questionnaire (Appendix A) comprised 15 questions designed to elicit both quantitative and qualitative data. The categorisations were aligned with the Office for National Statistics [37] for ease of analysis and validated interpretation. Simplicity in word choices was favoured in the questionnaire to counteract AP literature’s tendency towards techno-scientific jargon [23, 24]. For example, the word ‘clean’ was operationalised to evoke in the ‘subject’ the essence of an ‘object’ being free from adulteration. Similarly, ‘belief’ measured ordinary science intelligence and risk perception in everyday decision-making, meaning ‘accepting as true’ the factual status of something without necessarily comprehension or evidence [38]. The guided questionnaire went through an iterative process and was also tested among colleagues, with feedback incorporated, before being deployed.

### Participants and recruitment

All study participants were members of the public aged 18+, able to give consent and living within the ‘W’ postcode of West London (Appendix B). Recruitment was opportunistic and took place separately at individual sites, with prior permission from gatekeepers. Thus, S1 data came from a science fair event hosted by Imperial College London at the Kensington Campus; and S2 data came from a local community centre in White City offering various activities such as after school club, sewing group and free community lunch. Kensington is an affluent locale and White City is an ethnically diverse area with high density housing and pockets of significant economic deprivation and health inequalities.

### Data collection

All data was collected by the researcher during a one-week period in summer 2023. Prior permission to attend the events and collect data was obtained from events gatekeepers at both sites. Events visitors were then approached with invitations to participate in the study. No incentive was offered. Those who agreed were first screened for eligibility before completing the ethics procedure including providing a participant information sheet outlining the study purpose and a consent form to sign. Participants then proceeded to anonymously provide data in the form of digital voice notes. This data capture strategy was favoured because of its novelty, simplicity, and ease of engagement during busy interactive contexts whereby people’s typical engagement time is between five-ten minutes only, which characterised both sites. Furthermore, the ubiquity of digital technologies such as smart phones, have also rendered digital voice notes simple and quick to record; thus, they can facilitate efficient and quality data capture, with increased accuracy and integrity [39, 40].

To minimise potential for socially desirable responses, the researcher set up the digital recording device then stepped away from participants providing data. Participants were made aware that the study was not a test with right or wrong answers; rather, the study interest lay in their views, opinions and day-to-day experiences. Another key benefit of the researcher setting up the voice notes recorder was to ensure the correct placement of the microphone and to minimise unwanted background noise which can lower the quality of recordings and potentially impact the data interpretation [39].

Following our described study participant recruitment process, we distributed 40 questionnaires and retrieved 40 responses which in percentage terms translates to 100% response rate. This is because only those eligible and agreed to give their data were issued a guided questionnaire. Voice notes averaged one-four minutes and were transcribed verbatim. The final sample *n* = 40 comprise 14 (S1) and 26 (S2) participants. The study demographics is based on the events’ attendees at the time of data collection and those who accepted the researcher’s invitation to participate in the study. S1 event had a fixed end date, thus involved less data collection days; this was not the case with S2 thus providing more data collection days. This study was approved by the Imperial College Research Ethics Committee process (ICREC Reference Number 6642373).

### Data analysis

Quantitative data provided general demographic insights to facilitate a comparative analysis. Descriptive statistics of the brief questionnaires were calculated using R Statistical Package. Qualitative data was central to uncovering processes of HAP sense-making. Qualitative data was managed and analysed systematically following ‘The Framework Method’ [41]; which included data familiarisation, identifying a thematic framework, indexing, charting, and mapping and interpretation (Appendix C). This approach to thematic analysis of qualitative data was most suited to our study given the inherent explorative underpinnings of the phenomenon of HAP. As a tool, ‘The Framework Method’ is useful in shedding important light on a subject matter, to enhance contextual understanding [41]. Therefore, the emphasis on studies of this nature is not on statistical generalisation; rather detailed contextual understanding of the studied phenomenon, which in our study is HAP.

## Results

### Descriptive statistics

Table 1 provides a summary of the study sample (*n* = 40) demographic characteristics. S2 had more participants (65%) *n* = 40 compared to S1 (35%) *n* = 40; and with those aged under 50 more likely to fall in the latter group while those over 50 in the former. 70% of the sample identified as female; and 52.5% ethnically identified as ‘White’. Notably, the most discernible demographic difference between the two study sites were in education attainment and job/occupation. Examples of ‘technical skills’ included doctor, engineer, consultant, banker, architect, surveyor, and teacher; while ‘semi-skilled’ included community worker, hairdresser, and senior care assistant; and ‘unskilled’ workers included general gardener and cleaner.

**Table 1:** Demographics of the study participants at site one (S1) and site two (S2)

	Total (*n* = 40)	Site One (*n* = 14)	Site Two (*n* = 26)
Age			
18–29	7 (17.5%)	7 (50%)	0
30–49	15 (37.5%)	5 (35.7%)	10 (38.5%)
50–69	17 (42.5%)	2 (14.2%)	15 (57.7%)
70 and above	1 (2.5%)	0	1 (3.8%)
**Gender**			
Male	12 (30%)	5 (35.7%)	7 (26.9%)
Female	28 (70%)	9 (64.3%)	19 (73.1)
Non-binary	0	0	0
Prefer not to say	0	0	0
**Ethnicity**			
Asian or Asian British, Asian Welsh	5 (12.5%)	2 (14.2%)	3 (11.5%)
Black, Black British, Caribbean, African, Black Welsh	6 (15%)	2 (14.2%)	4 (15.4%)
Mixed or Multiple group	3 (7.5%)	0	3 (11.5%)
Other ethnic group	5 (12.5%)	0	5 (19.2%)
White	21 (52.5%)	10 (71.4%)	11 (42.3%)
**Education Attainment**			
Primary School	3 (7.5%)	0	3 (11.5%)
GCSE or Diploma	17 (42.5%)	2 (14.2%)	15 (57.7%)
Bachelor’s Degree	11 (27.5%)	6 (42.8%)	5 (15.2%)
Master’s degree and above	9 (22.5%)	6 (42.8%)	3 (11.5%)
**Health Condition (Self)**			
Asthma	5 (12.5%)	0	5 (19.2%)
Heart Disease	2 (5%)	0	2 (7.7%)
Other	4 (10%)	1 (7.1%)	3 (11.5%)
Missing and /or not provided	9 (22.5%)	7 (50%)	2 (7.7%)
No Health Condition Reported (N/A)	20 (50%)	6 (42.8%)	14 (53.8%)
**Health Condition (Carer)**			
Asthma	4 (10%)	0	4 (15.4%)
Heart Disease	2 (5%)	1 (7.1%)	1 (3.8%)
Other	0	0	0
Missing and /or not provided	22 (55%)	8 (57.1%)	14 (53.8%)
No Health Condition Reported (N/A)	12 (30%)	5 (35.7%)	7 (26.9%)
**Job / Occupation**			
Skilled / Technical	17 (42.5%)	12 (85.7%)	6 (23%)
Semi-skilled	6 (15%)	0	6 (23%)
Unskilled	7 (17.5%)	0	7 (26.9%)
Retired	4 (10%)	0	4 (15.4%)
Unemployed	3 (7.5%)	0	3 (11.5%)
Student	2 (5%)	2 (14.2%)	0
**Reason for attending event**			
Supporting friend’s Exhibition	6 (15%)	6 (42.8%)	0
Passed by / Was in vicinity	2 (5%)	2 (14.2%)	0
Direct interest / Event seeking	32 (80%)	6 (42.8%)	26 (100%)

### Emerging themes

**Fig. 1:**
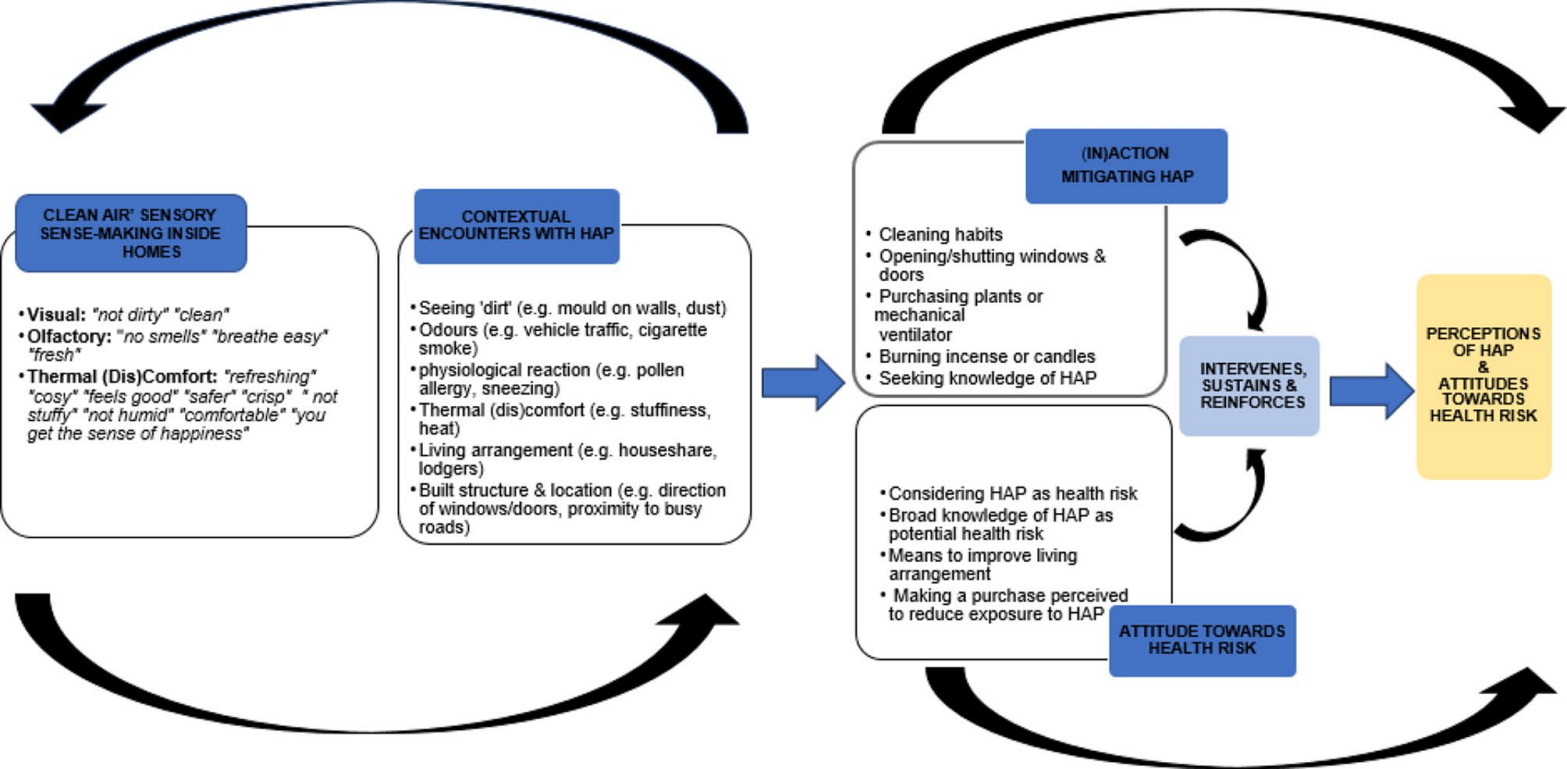
Schematic diagram explaining processes of IAQ sense-making, interpretation and knowledge formation resulting in perceptions of HAP and attitudes towards health risk

Three key patterns emerged from the data and were identified as core themes associated with ‘knowing’ HAP. They are: *Stimulative Effects*, *Contextual Conditions*, and *Risk Attitudes*. Within *Stimulative Effects* and *Contextual Conditions*, a further three sub-themes were identified. Together, they provide a more holistic understanding of participants’ knowledge formation of perceptions of HAP and attitudes towards AP as a health risk (Fig. 1). These are elaborated further in the next section.

#### Stimulative effects

In this study, ‘stimulative effects’ are indicative of how study participants initially experienced clean air and were subsequently motivated to consider HAP. Descriptions from participants illustrated understandings, sense-making, and meaning-making of HAP as contextually bound, subsequently, resulting in distinctive clean air knowledge formation [20, 24, 31]. Interactions with ‘stimulative effects’ was the most prevalent manner for making sense of IAQ within the home setting. This was followed by the ‘triggering’ of specific actions or consideration of IAQ. These ‘triggering’ events were further analysed, and which uncovered three explanatory sub-themes including a) *Sensory perception*,* b) Built structure and location*,* and c) Health condition or broad knowledge of AP health impacts.*

- **Explanatory sub-themes**
  - *Sensory perception* The data revealed that the most prevalent mode of awareness or concerns for HAP was directly through sensory perception. This includes visual, smell and thermal (dis)comfort. Responding to the first question: “*What does clean indoor air feel like to you?”*, participants stated a mix of words, descriptive contexts and metaphors illustrating their understanding of the notion of clean air. Common examples included “*not dirty” “refreshing” “no smell” “clean” “comfortable” “safer” and “cosy”.* Additionally, sensory perceptions were characterised positively or negatively, which suggests affective awareness. For example, responses to: *Which room in your house do you feel has the cleanest air and why?* included “*My room*,* it has a nice bay window which lets in fresh air”* (Participant 14, S2) and *“Kitchen…err…bathroom because its furthest from street and feels cleaner….”* (Participant 12, S1).
  - *Built structure and location* The second most prevalent HAP awareness mode and meaning making was through the home itself, as a built structure, and its location. Several participants articulated initially noticing a specific anomaly (e.g. smelling odorous fumes from vehicle traffic, seeing dust, or mould stains). This triggered concerns of IAQ impacts within their homes, leading some to take specific perceived remedial action. Prominent features of concern included the presence of windows to allow ventilation (including position in built structure), proximity to roads and limited indoor space. This was particularly evident in S-Two data, where examples were drawn from lived experience. For example, when we asked: What do you do to keep the air inside your home clean? one response was: *“Do cleaning*,* I always clean it all the time. …washing*,* spraying with stuff from the supermarket…It’s because I live West Way…air is no fresh…I don’t feel good*,* I have bronchitis. I live too many years*,* 20 years.”* (Participant 35, S2).
  - *Health condition or broad knowledge of AP health impact* The final ‘trigger’ for HAP awareness and meaning making was having a health condition or broad knowledge of poor health outcomes associated with AP. Participants ‘without a health condition’ formed 50% of the sample, while 30% reported to ‘care for’ (carer role) someone with a health condition and which asthma was most prevalent, followed by allergies. While those with direct experience (self or carer) mentioned specific health conditions as their reason for noticing and making sense of IAQ within their home, others broadly mentioned impacts on health and wellbeing such as respiratory conditions and hay fever. For example, when we asked: Do you think your health can be affected by poor indoor air quality? (Answer Yes or No and Why*)*, majority 80% responded ‘Yes’, 10% ‘No’ and 10% unsure. It was unclear to what extent the sample majority is directly or indirectly informed (self or carer role) as most S1 participants either did not provide this data point or declared no existing health condition. Some of the responses obtained included: “*Yeah*,* of course…when I work in a plaster area or a clay area*,* which is not well ventilated*,* I feel like my nose get blocked. I have runny nose or I cough a lot and I feel stuffed inside.”* (Participant 12, S1), *“I don’t think …because you keep the place clean.”* (Participant 38, S2), and *“Um…it’s not something I’ve ever considered*,* but I guess in a different way…yeah. Purely because of things lingering more than anything else… This is the first I’ve heard of this concept.”* (Participant 14, S1).

#### Contextual conditions

The data revealed specific contexts underpinning participants’ knowledge of HAP. These included characterisations of distinct events where participants encountered HAP in their day-to-day. Subsequently, participants were either driven to address the issue because of implication concerns, or any perceived and/or imagined constraints in their personal circumstances became apparent. Notably, ‘contextual conditions’ were commonly provided as elaborations and/or explanations to specific (in)actions. These were categorised into three sub-themes including a) *Recurring encounters*,* b) Living situation and arrangement*,* and c) Other sources of IAQ knowledge.* For example, response to: Do you believe you can improve quality of air inside your home? (Answer Yes or No and give example How) elicited 57.5% ‘Yes’. All S1 participants fully agreed, with majority providing examples, and only 0.28% were unsure how. For S2, 50% disagreed while 50% partially agreed, and some provided examples.

- **Explanatory sub-themes**
  - *Recurring encounters* The data revealed recurring encounters which underpinned interpretations of the notion of clean air. Notable examples included seeing mould on walls or dust on surfaces; smelling both cooking aroma and odorous vehicle traffic fumes; and feelings of ‘stuffiness’ in the air. Elaborations include: *“ Yes*,* I like cooking smells they make the place lovely and homely.”* (Participant 49, S2); and *“… I dunno why the mould coming inside my house. Always I am cleaning everywhere very good but I dunno why…. [Housing Estate Officer] need to think why the mould coming…but they tell me I need clean and open the window…”* (Participant 19, S2).
  - *Living situation and arrangements* Contexts of shared accommodation and its limitation was commonly described, through participants’ personal experiences, as impacting their abilities to improve IAQ within their homes. Participants’ responses largely portrayed feelings of lacking means and ‘own ability’ to change their living arrangements. The following examples demonstrate S2 participants’ responses relating to lived experience, while for S1 they appeared broad and generic:
    *“…I’m of Asian background so we use agarbatti*,* it is… incense!… to keep the place clean… if you can’t open doors and windows incense is the one that keep the air fresh instead of artificial room fresheners….”* (Participant 36, S2); *“…people still smoke* [in their home] *this is my situation with my landlord but I am just a lodger so what can I do? I start to cough then I go out…* [for] *fresh air… I keep my room fresh with good smells…even smoking smells can be removed with good air freshener…”* (Participant 46, S2); and *“Yes*,* find a way of creating consistent natural airflow in rooms …like keeping doors*,* windows open or even air purifying filters…”* (Participant 7, S1).
  - *Other sources of IAQ knowledge* Participants revealed some of their sources of IAQ knowledge in responses to: Have you ever come across information on how to improve indoor air quality? (Answer Yes or No). ‘Yes’ elicited 35% with majority of this (80%) coming from S1. Majority S2 responded ‘No’ and only 7.7% of the entire sample could not recall. Specific sources of other IAQ knowledge were revealed when we asked participants: Do you believe you can improve quality of air inside your home? (Answer Yes or No and give example How), responses included *“Yeah*,* I heard about…some very famous like Dyson Purifiers or competitors… but I am quite aware about the electrical… could release some plastic chemicals in the air.”* (Participant 9, S1); *“…kind of… because it’s something I’m interested in. I’ve seen things on Twitter and read articles on filtration*,* but I’ve never come across any public health information about improving indoor air quality…that’s something I would really welcome….” (*Participant 2, S1); *“No*,* not really…But the housing authority repair guys tell us to open the bathroom windows to stop mould growing on walls.”* (Participant 46, S2); and *“No*,* not really but there is always news about pollution from cars.” (*Participant 47, S2).

#### Risk attitudes

Uncovering the building blocks of participants’ attitudes towards health risks associated with HAP forms the final core theme identified in the data. Responses to: Do you think indoor air pollution can be worse than outdoor air pollution? (Answer Yes or No), overall, ‘Yes’ elicited 35%, with S1 more likely to agree. Here, data also showed that, unlike S1 participants, S2 participants tended to anchor their response in lived experience. Notably, S2 participants’ ‘Yes’ responses referenced smoking habits as a reason and ‘No’ responses referenced location of homes along busy trafficked road/motorway as reasons. For example: *“Um…I didn’t think so. So*,* no…”* (Participant 5, S1); *“No. Absolutely not. Because when you close the door*,* you left alone… because I don’t open the door*,* the window or nothing.”* (Participant 18 (S2); and *“Um…It could be…although I don’t think it’s the case in my house*,* but it could… definitely if it is not well ventilated. Yeah. And if there’s…humidity and mould on the walls.”* (Participant 10, S1).

## Discussion

The results deliver context regarding how individuals formulate their knowledge and understanding of HAP. Sensory perception was identified as primarily motivating participants to ‘make sense’ of IAQ within their homes. While visual and olfactory senses were consistently revealed as key underpinnings of stimuli to consider IAQ, thermal (dis)comfort was also evident. Other factors included the house itself as a built structure and/or its location, the presence of a health condition and broad knowledge of AP health impacts. Moreover, such consideration happened within distinctive contexts inside homes resulting in sense-making and evaluations for subsequent (in)action. It was through this process that perception of HAP was formed, and ultimately, illustrated the building blocks of attitudes towards health risk. Because sense-making is fundamental to this study, the use of ‘place’ as a conceptual guiding tool was critical to centring individuals, their goals and desires. This tool also facilitated the analysis of subsequent (in)action within multiple ‘micro geographies’ of participants’ homes where forms of pollution generating activities can happen at any time. Through this process, it is possible to contextually understand participants’ rationale and appreciate subjectivities in interpretations of what constitutes ‘pollutants’ inside homes. Adding to previous work, this study can facilitate effective and meaningful public health HAP risk communication strategies.

### Re-animating HAP

#### Knowing ‘clean air’

Our results are aligned with research reinforcing the significant role of lived experiences in shaping an individual’s understandings of AP [20, 22, 24]. Additionally, experiences of AP are coproduced alongside context, space, and time [30, 31, 34, 36]. Participants expressed understandings of IAQ within their home setting predominantly in relation with bodily senses. Visual and olfactory evidence was most powerful in mediating HAP knowledge, followed by thermal (dis)comfort. Most participants described ‘cleaning’ activities, by way of removing visually seen ‘*dirt*’ (pollution) as how they perceived ‘clean air’ inside their homes. While ventilation practices (e.g. opening doors-windows) were also mentioned as good ways to maintain IAQ through letting in ‘*fresh air’* or *‘cool breeze’*, cleaning activities (e.g. using supermarket products to clean surfaces, washing mould off walls and spraying to neutralise odours) were most prevalent. The common removal of dirt through cleaning suggests a preference for ‘seeing things in place’ (e.g. orderliness) as opposed to ‘out of place’ (disorderliness) according to individual interpretation. Thus, physical manifestation of *‘dirt’* is considered visually ‘out of place’ inside the home and must be removed. For many participants, physical sightings of ‘pollution’ inside homes is portrayed as a transgression of personal space boundary. A place considered *‘clean’*,* ‘not dirty’ and ‘cosy’* thus, is *‘safe’* and provides protection from polluted ‘outdoors’. As such, attention is paid to maintain this order, through habituated ‘*cleaning with stuff from the supermarket*’ and spraying aerosol fragrances as ‘*even smoking smells can be removed with good air freshener smells’*.

However, a recent meta-analysis [9] on the human health effects of commonly used products to chemically clean and disinfect surfaces inside residential and public buildings found an increase of exposure to a variety of chemicals and particulate matter that pollute indoor air and particularly has negative effects on human respiratory health. 51% of the studies reviewed were of household exposure to cleaning chemicals, and which the conclusion was that frequent use of cleaning products in indoor contexts and poor ventilation practices may increase occupants’ exposure to a variety of harmful environmental toxicants including volatile organic compounds (VOCs), particulate matter (PM) and nitrogen dioxide (NO₂). The study also found that people who habitually handle or are in close contact with such products have an elevated risk of asthma and rhinitis. Another study [10], also evaluating cleaning products and regular usage, assessed the daily indoor learning environment of school children in France and found a significant complexity of chemicals and other substances contained in regularly used cleaning products. These were in form of, for example, fragrance, stabiliser and sanitiser. Additionally, the study found the product contents also classified as harmful, toxic or irritant under the European Union Directive regulating the classification, packing and labelling of dangerous substances. A prior study [13] simulated conditions that reflect common day-to-day encounters in many indoor environments occupied by office workers, including exposure to VOCs which are a variety of chemicals that pollute the air and can cause both short and long-term adverse health effects. A key finding was that VOC exposure does have a negative impact on cognitive functions, with the control group scoring 61% higher than exposed group.

In our study it may be that participants are perceiving or interpreting ‘clean air’, ‘ways to maintain IAQ within home settings’ and ‘HAP affecting health’ in terms of hygienically washing and the broad removal of visual representations of ‘*dirt*’. This underscores the importance of understanding context as such is indicative that HAP perception depends on a specific frame of reference and is inherently value laden. This view is aligned with findings from a study [42] that suggested four key factors as influencing individuals’ behaviours which are environmentally significant, in this case HAP. They are (a) individual attitudes, values and beliefs which are linked to environment, but weighted alongside considerations including comfort, aesthetics, and quality; (b) contextual drivers including social, economic, institutional, and political specificities; (c) individual capabilities in relation to knowledge, skills and resources; and finally (d) habituated practices. All these factors underscore individual perceptions towards HAP. Our study insight into how people ‘know clean air’ is also consistent with an empirical study [43] on hospital cleanliness in West Midlands, UK, which found that by improving public perception of hospital cleanliness, through visual and olfactory evidence (e.g. nurses handwashing, ambient smell of disinfectant), the public’s ‘subjective’ understanding of hospital cleanliness improved. Additionally, this had the effect of improving public perceptions of the hospital’s rate of health-care associated infections (HCAIs), such as Norovirus, and which the study participants were broadly unaware of the symptoms.

#### Cultural sense-making of ‘clean air’

Our findings highlighted perceptions that occasional scented candle-burning improves IAQ within home settings, just as the perception that ‘*even smoking smells can be removed with good air freshener’.* This raises important questions around cultural interpretations of clean air. An especially key insight came from a participant who self-identified as culturally Asian and prone to coughs, congestion and allergies including ‘hay fever’. They stated that they habitually burn ‘*agarbatti’* (incense) for ‘*fresh air’* and described this practice as a culturally normative way to ‘*clean the home’*, to compensate for ventilation constraints. This practice suggests a fundamental misconception of what constitutes ‘pollutants’ given the significant health risks associated with incense-burning inside homes [11]. Implied ‘scientific objectivity’ in understandings of AP has previously been critiqued [31], on the basis that AP does not exists ‘out there’, independent of society, individuality, or culture. Thus, the cultural practice of incense-burning, illustrated in our study, is evidence that scientific and cultural forms of AP knowledge maintain fundamentally different epistemologies. This insight underscores the importance of contextual understanding of a phenomenon, to better appreciate rational underpinnings of a particular action.

#### Socioeconomic status and ‘clean air’

The results provided important insights around possible health impacts of HAP. For example, in contemplating IAQ, a S2 participant, whose home location is along a heavily trafficked motorway, opined *“…maybe if I bought the machine*,* my friend*,* he bought…*[dehumidifier]…*but it’s too expensive and use too much electricity…”*. Their response to the possibility of IAQ being worse than outdoors was a vehement “*No. Absolutely not. Because when you close the door*,* you left alone… I don’t open the door*,* the window or nothing.”* This participant’s compensatory action was to restrict ventilation, having already confronted their lack of economic self-efficacy to improve their IAQ through a purchase. To this participant, sensory evidence of ‘*pollution*’ manifesting visibly (dust), audibly (noise) and odorously (fumes) is more significant than scientific measures such as PM, NOx, NO^2^ etc [5, 7]. The participant’s rationale in seeking to be “…*left alone* …” when they “…*close the door…”* is consistent with an insect infestation ‘nuisance’ case study [44] in Sweden whereby residents’ analysis and solutions were coherently anchored in lived experiences, as opposed to scientific evidence. For study participants, ‘pollution’ was constituted by their corporeal and sensory HAP encounters, not necessarily PHE’s summary public-facing informational guidance that describes pollution as ‘*invisible’* or not necessarily viscerally discernible.

Therefore, such compensatory action sheds light on important, yet ‘invisible’, day-to-day encounters with HAP. Common public health interventions may be overlooking these distinctive constraints to IAQ improvements within home settings. Historical debate [33] on the production of natural scientific information has heavily critiqued the practice as being prone to distilling and filtering out the importance of human’s connectivity with ‘place’; and more recent analysis [20] emphasises the importance of interdisciplinary research tools in providing a comprehensive understanding of environmental health patterns observed in quantitative-focused studies. Notably, our study finding in relation to socioeconomic status and ‘clean air’ suggests important links between low socio-economic status and the awareness of HAP mitigation barriers. This is consistent with other studies [22, 24] that have shown a lack of self-efficacy (perceived or real) as a barrier to the adoption of scientific advice on AP exposure mitigation; with particular emphasis [22] on increased personal representativeness of AQ data as key to addressing AP mitigation barriers individuals face.

Our findings furthermore illustrate that low self-efficacy around improving IAQ was prevalent among S2 participants, as well as among those who self-described as unskilled (26.9%), unemployed (11.5%) and/or retired (15.4%). Overall, 85.7% of S1 participants self-described as skilled technically, compared to 23% of S-2. Given that S1 was a science context, there may be a potential for self-selection in attending the event which reflects the high technical skills reported. GCSE (General Certificate of Secondary Education) or Diploma was the sample’s most common education level attainment (42.5%), and S2 comprised the majority (57.7%). This insight confirms S2 locale’s association with economic deprivation and low educational attainment among residents. In contrast, S1 locale is economically one of the wealthiest addresses in the UK. While London post code ambiguity is noted, a UK study [45] on AP suggest areas with the highest deprivation levels in London, on average, also have the highest levels of AP. Similarly, a study in Belgium [46] found income levels to be a significant predictor of AP and noise exposure among the city residents, with those of low socio-economic status experiencing increased exposure. Thus, recognising housing as a social determinant of health [47, 48] is vital to national strategies for action to address HAP as a health risk. As England’s CMO in the 2022 AP report [15] notes, preventing and reducing indoor AP should now be a priority.

#### Other ways of ‘knowing’ pollution

AP perception studies suggest that ‘meaning’ and ways of articulating pollution, such as ‘severity’, is strongly mediated by ‘media/press’ [23, 24, 49]. In our study, participants’ responses suggest a general knowledge of HAP-related health impacts by, for example, referencing seeing on “…*the news…”*, or “…*videos on YouTube showing houses covered in mould…”.* An important difference is that while S1 participants demonstrated general awareness and observations about pollution, S2 participants described lived experiences. It was not possible to discern the extent of ‘other’ influences on perceptions of HAP, beyond evidence of some engagement, even if only passively. Additionally, most participants referenced outdoor AP to anchor their views on indoor AP when making sweeping observations such as “…*many people driving cars and causing pollution”.* Nonetheless, some participants’ detailed descriptions may demonstrate a level of awareness or engagement with knowledge on poor IAQ and potential health impacts. This underscores the importance of easily accessible information in relation to spill over effects from distinctive recurring HAP-related challenges.

Another important pattern our study found is in relation to ‘knowing’ pollution through the presence of health condition. This was not the most common motivator for ‘knowing’ pollution [24]. This finding suggests that having an existing health condition, such as asthma, did not appear as primary stimuli for individuals to spend personal time considering or seeking information about HAP, or improving their IAQ. Even participants who disclosed a health condition, which was mostly S2, still discussed HAP in relation to remedial ‘*issues inside homes’* or ‘*from traffic outside’*. It may be that wider social ‘issues’ among this study population are more prominent in their day-to-day, consequently, taking centre stage in individuals’ lives. Or it may be low awareness of possible association between poor IAQ within their home setting and poor health outcomes [14]. The majority of S1 participants either self-described as having ‘No’ health condition (self or carer) or did not provide this data.

### Health Risk Perception: asymmetry of actions and intentions

Consistent with AP perception studies [20, 24, 30, 31], our study found that the idea of risk is socially negotiated based on individuals’ lived experience, values, as well as trust in institutions [50, 51]. Experiential thinking [51] or accessibility [50], used by Slovic (2000) and Kahneman (2003), respectively, help explain how, through habituated ‘references’, our study participants perceive HAP health risk. Both scholars also underscore contextual sensory stimuli, especially visual, in knowledge formation. The social dimension of food preparation and cooking practices can provide such experience viscerally. Cooking is a common HAP source through the generation of harmful gases and particulates, and which the risk to health is cumulative [3, 6, 8]. While few participants in our study mentioned concurrently ventilating, many responses suggested perceptions of ‘cooking smells’ only in terms of making the *‘place lovely and homely*’ because *‘it’s only good smells’*. However, evidence shows significantly high peak (cooking times) ultrafine particulates (UFP) concentrations of 14 times the average indoor-to-outdoor UFP in poorly ventilated homes [8].

Thus, observing from the perspective of experiential thinking [51] or accessibility [50], the cumulative risk of cooking practices is invalidated through participants’ experiences of associating the practice with ‘*only good smells*’ (reference-dependent pleasant social context). This is opposed to ‘scientific objectivity’ where cooking practices are known to generate HAP, and which the risk to health is cumulative [8]. Kahneman argues, key attribute of perception is its inherent role of magnifying differences, to create a ‘reference’ for the next time that detail is ‘recalled’. While in normative social dimensions it can be proportionate to appreciate pleasant cooking ‘*smells’*, at once by masking, the activity renders ‘invisible’ the dangers of harmful pollutants also being generated, particularly through processes of combustion. Moreover, within distinctive ‘reference’ of making the *‘place lovely and homely*’ (context), such is grounds for powerful combination of ‘Stimulative Effects’ which comes to underpin sense-making in relation to HAP perception and forms the building blocks of attitudes towards health risk. This is in line with findings from other evaluative studies [50, 51] that suggest risk perceptions is derived from day-to-day, localised and situated sensations embedded within a social-cultural dimension.

Considering this insight, public health communication of cooking activity in terms of HAP and health risk is both substantially challenging and yet an imperative. Our study findings show a strong reliance on lived experience of sensory perceptions to assess HAP health risk. *‘Cooking smells’* are clearly in contrast to offending odours (e.g. cigarette fumes), which is unwelcomed as a transgressor of both bodily senses and home as ‘place’; and may trigger a physiological reaction such as sneezing which can be a vital AP cue. However, sensory perception alone is insufficient and unreliable in understanding the nature of cumulative risk [50, 51], such as health-associated HAP, which can potentially be masked by *‘lovely and homely cooking smells’*. This indicates that there are possible epidemiological risks associated with principally relying on sensory responses to assess IAQ within home settings. Not only do the more insidious HAP (e.g. UFP and PM) escape sensory detection at average indoor concentration levels [8], but some pollutants occupy different structural forms including lethal ones like carbon monoxide (CO), which is a colourless, odourless, and tasteless gas [52]. Thus, with HAP largely rendered ‘invisible’ in repetitive day-to-day activities, especially the highly habituated practice of cooking, so is the extent of health risk perception inside participants’ homes.

### Strengths and limitations

This empirical study stands out for its early exploration of HAP in the UK, using mixed methods in the traditionally quantitative field of environmental health epidemiology. Guided by a conceptual framework, this study contextualises ways in which individuals’ knowledge and understanding of HAP is formulated and how these become the building blocks of attitudes towards health risk. Sensory perception is identified as primarily motivating participants to ‘make sense’ of IAQ within their home setting. Findings demonstrate the importance of social and geographical context in HAP evaluation and sense-making. However, this study has limitations. First, the sample may not be representative of West Londoners in terms of views and opinions of HAP which were self-reported through a survey, potentially limiting generalisability. Second, recruitment was based on participant attendance of events at both sites and which the impact may be self-selection. Study majority self-identified as female and S2 participants comprised sample majority. Finally, given limited research in HAP, part of the discussions necessitated drawing parallels with perception studies on AP broadly, to enhance understanding of sense-making and health risk analysis processes.

### Recommendation for policy makers

#### Decentralised risk communication efforts

Shift away from conventional top-down model of AP policies on risk communication; efforts and resources to address HAP must be brought closer to communities, where latent knowledge exists within local authorities about residents’ day-to-day challenges and dwelling types, which can inform more effective strategies.

#### Reduced individual burden

Avoid placing the full responsibility of controlling HAP ‘source and exposure’ solely on individuals. Our study reveals, the majority who appear severely impacted by HAP and would most benefit from risk communication efforts, are simultaneously dealing with a range of other day-to-day challenges which policy considerations may be omitting.

#### Address knowledge gaps

Misconceptions about pollutants and mitigation strategies persist particularly among those who are more affected by HAP. Risk communication efforts should address these gaps to improve public health outcomes.

#### Refine indoor air quality (IAQ) measurement protocols

IAQ measurement protocols should incorporate individuals’ lived experience; this would complement objective air quality data with insights from residents’ sensory perceptions which often differ from standard measurements.

### Conclusion

Focused on understanding individuals’ perceptions of ‘clean air’ inside homes across West London, this mixed-methods study adopted a comparative approach in assessing two different social contexts. The aim was to investigate underpinnings of views on indoor AP, and the influence of contextual factors on perception. By uncovering these insights, the study has contributed to understanding the foundational elements shaping attitudes towards health risk associated with indoor AP within residential settings.

Findings revealed three key themes in relation to HAP sense-making: (1) *Stimulative Effects*, (2) *Contextual Conditions*, and (3) *Risk Attitudes*. As *stimulative effects*, sensory perceptions (vision, olfactory evidence and thermal (dis)comfort) was identified as primarily motivating participants to ‘make sense’ of IAQ. This happened within specific *contextual conditions* inside homes, where individuals were motivated to consider/make evaluative decisions for subsequent (in)action. Through this process, participants interpret and form their knowledge of IAQ; ultimately, developing their perceptions of HAP and health *risk attitudes*. Study insights fundamentally show a strong reliance on lived experience of sensory perceptions to assess HAP and health risk. For example, pleasant ‘cooking smells’ or the cultural practice of incense-burning are welcomed in contrast to offending cigarette odour which is unwelcomed as a transgressor of both bodily senses and home as ‘place’. Similarly, habituated cleaning practices using common supermarket products is perceived to remove pollution from the home, while limiting ventilation is considered as providing protection from polluted ‘outdoors’. However, all these day-to-day activities and behavioural practices are common sources of cumulative HAP which can be harmful to health, particularly respiratory health. We argue that reliance upon sensory cues to determine what constitutes ‘clean air’ is invariably subjective and is characterised by distinctive contextual factors (‘place’). These factors create a perception gap and pose risk to health. Such is particularly exemplified in the case of cooking and incense-burning which individuals highly enjoy and value; likewise, in the day-to-day use of chemical and disinfectant cleaning products to ‘remove pollution’ from their homes.

Therefore, in line with the 2022 CMO report on AP stating the importance of indoor AQ research through understanding prevention and mitigation, our study findings demonstrate an imperative of understanding contexts in which individuals encounter HAP. This may facilitate strategies in developing effective and meaningful public health policies and interventions on HAP.

This study highlights the fundamental value in interdisciplinary efforts to understand how AQ inside homes can contribute to improved public health. Future research in HAP would benefit from adopting and designing mixed-methods studies, with a focus on co-production and a larger sample size.

## Electronic supplementary material

Below is the link to the electronic supplementary material.

Supplementary Material 1: Study guided questionnaire

Supplementary Material 2: West London district W postcode

Supplementary Material 3: Framework analysis for qualitative data

## Data Availability

The data generated during this study are not publicly available due to confidentiality reasons and ethical restrictions but are available from the corresponding author on reasonable request.

## Abbreviations

AP: Air Pollution
AQ: Air Quality
IAQ: Indoor Air Quality
HAP: Household Air Pollution
