# Supplementary material for: ‘Incense is the one that keeps the air fresh’: indoor air quality perceptions and attitudes towards health risk
Source: BMC Public Health. 2024 Nov 14;24:3178. doi: 10.1186/s12889-024-20635-1 (PMC11566293; doi:10.1186/s12889-024-20635-1)
Supplement: Supplementary file 1 — Supplementary Material 1: Study guided questionnaire [file 12889_2024_20635_MOESM1_ESM.pdf]

Guided Questionnaire

- ***Views and opinions:***

1. What does clean indoor air feel like to you?

2. Which room in your house do you feel has the cleanest air and why?

3. What do you do to keep the air inside your home clean?

4. Do you think indoor air pollution can be worse than outdoor air pollution? (Answer Yes or No)

5. Do you believe you can improve quality of air inside your home? (Answer Yes or No and give example How)

6. Have you ever come across information on how to improve indoor air quality? (Answer Yes or No)

7. Do you think your health can be affected by poor indoor air quality? (Answer Yes or No and Why)

- ***Demography:***

Please **tick** this ☐ box to **confirm** you live in West London and your postcode begins with the letter 'W' (for example W1, W2, W3, W4, W6, W8, W9, W10, W11, W12, W14)

Age

8. Which of the following best describes your age? Select one.

▪ 18-29 ▪ 30-49 ▪ 50-69 ▪ 70 and over

Gender

9. Which of the following genders do you most identify with? Select one.

▪ Male ▪ Female ▪ Non-binary ▪ Prefer not to say

Ethnicity

10. Which of the following best describes you? Select one.

▪ Asian or Asian British, Asian Welsh ▪ Black, Black British, Caribbean, African, Black Welsh
▪ White ▪ Mixed or Multiple groups ▪ Other ethnic group

Education

11. What is the highest level of education you have completed? Select one.

▪ Primary school ▪ GCSE or diploma ▪ Bachelor's degree ▪ Master's degree and above

Health

12. Do you have any of these health conditions or another respiratory condition (write N/A if you don't)

▪ Asthma ▪ Heart Disease ▪ Other respiratory condition (please name) …………….………………………………………

13. Do you care for anyone with these conditions or another respiratory condition (Write N/A if you don't)

▪ Asthma ▪ Heart Diseases ▪ Other respiratory condition (please name) ………………………………………………..

Job/ Occupation

14. What job or work do you do, and which industry best fits this job or work? (Write N/A if you don't work)

……

Attending Event

15. What is your reason for attending this event?...

Thank you very much for taking the time to complete these questions.
