# Supplementary figures and images for: ‘Incense is the one that keeps the air fresh’: indoor air quality perceptions and attitudes towards health risk
Source: BMC Public Health. 2024 Nov 14;24:3178. doi: 10.1186/s12889-024-20635-1 (PMC11566293; doi:10.1186/s12889-024-20635-1)

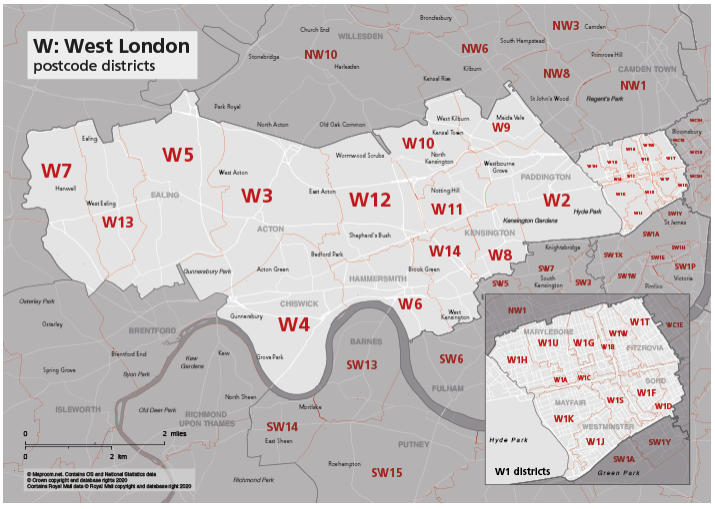

Supplement: Supplementary file 2 — Supplementary Material 2: West London district W postcode [file 12889_2024_20635_MOESM2_ESM.png]

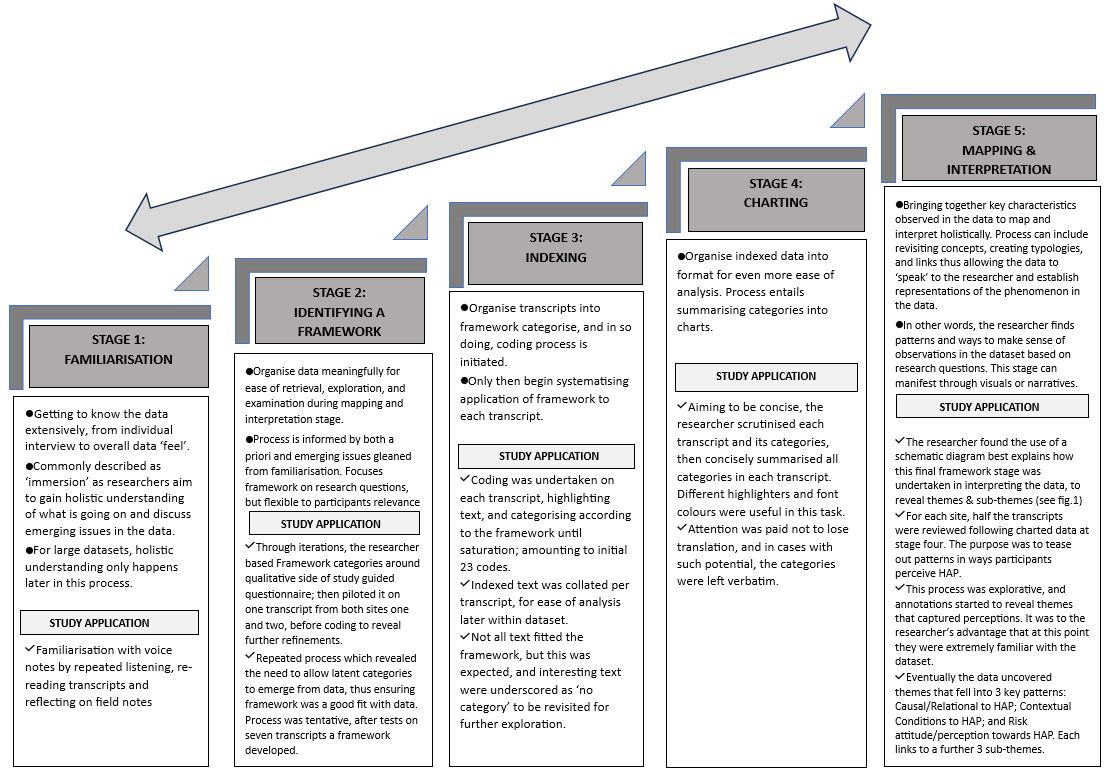

Supplement: Supplementary file 3 — Supplementary Material 3: Framework analysis for qualitative data [file 12889_2024_20635_MOESM3_ESM.png]
